# Microencapsulation of Phosphorylated Human-Like Collagen-Calcium Chelates for Controlled Delivery and Improved Bioavailability

**DOI:** 10.3390/polym10020185

**Published:** 2018-02-14

**Authors:** Yu Mi, Zhengfang Liu, Jianjun Deng, Huan Lei, Chenhui Zhu, Daidi Fan, Xingqiang Lv

**Affiliations:** 1 Shaanxi Key Laboratory of Degradable Biomedical Materials, School of Chemical Engineering, Northwest University, 229 North Taibai Road, Xi’an 710069, Shaanxi, China; mi_yu@nwu.edu.cn (Y.M.); zhengfangliu89@163.com (Z.L.); dengjianjun@nwu.edu.cn (J.D.); 201520729@stumail.nwu.edu.cn (H.L.); lvxq@nwu.edu.cn (X.L.); 2 Shaanxi R&D Center of Biomaterials and Fermentation Engineering, School of Chemical Engineering, Northwest University, 229 North Taibai Road, Xi’an 710069, Shaanxi, China

**Keywords:** alginate, chitosan, microcapsules, PHLC-Ca, bioavailability

## Abstract

The bioavailability of Phosphorylated Human-like Collagen-calcium chelates (PHLC-Ca) as calcium supplement is influenced by the extremely low pH and proteolytic enzymes in the gastrointestinal tract. This study addresses these issues by microencapsulation technology using alginate (ALG) and chitosan (CS) as wall materials. The different ratio of ALG to PHLC-Ca on microcapsules encapsulation efficiency (EE) and loading capacity (LC) was evaluated and 1:1/2 was selected as the optimal proportion. The microcapsules were micron-sized and spherical in shape. PHLC-Ca was successfully entrapped into the matrix of ALG through forming intermolecular hydrogen bonding or other interactions. The confocal laser scanning microscopy (CLSM) indicated that CS was coated on ALG microspheres. The MTT assay exhibited that CS/ALG-(PHLC-Ca) microcapsules extracts were safe to L929. The animal experiment showed that CS/ALG-(PHLC-Ca) microcapsules was superior to treating osteoporosis than PHLC-Ca. These results illustrated that the bioavailability of PHLC-Ca was improved by microencapsulated.

## 1. Introduction

Calcium deficiency is one of the major nutritional deficiencies in the world which can cause osteoporosis, hypertension, colon cancer, and kidney stones [1]. Although food contains calcium, some studies have showed that most people would not get enough calcium from their diet [2]. Thus, many investigators have concentrated on the development of various calcium supplements with reasonable calcium bioavailability such as calcium gluconate, amino acids-calcium and calcium-binding peptide [3,4,5,6,7,8,9]. Although the calcium content of those calcium supplements is high, their low absorption rates and poor bioavailability impede their wide use as calcium supplements. As the bone health is closely related to protein, calcium, and phosphorus intake, the existence of protein may improve the solubility and bioavailability of calcium [10]. In recent years, to avoid the deficiency of metal trace elements, the chelates formed by protein and metal ions have been investigated as a potential approach to delivering a variety of microelement of the body need at a required quantity [11]. Human-like collagen (HLC) was obtained from recombinant Escherichia coli containing human-like collagen cDNA [12]. HLC has several special characteristics such as water-solubility, nontoxic, biocompatibility and biodegradability [13,14] and has been used in inartificial bone, vascular scaffolds and novel hemostatic materials [15,16].

Phosphorylated Human-like Collagen-calcium chelates (PHLC-Ca) were prepared in our previous studies plays an active role in anti-osteoporosis [17]. However, protein calcium supplements have their limitations used as protein drugs in oral administration [18,19]. PHLC-Ca is easy to inactivation or degradation under the extremely low pH and proteolytic enzymes in the gastrointestinal tract, which would result in the low bioavailability upon oral administration. Microencapsulation technology represents a promising strategy to solve these problems [20,21,22,23].

Alginate (ALG), a water-soluble natural anionic linear polysaccharide, is used as a pH-sensitive material for the microencapsulation of protein and peptide drugs and enables encapsulated drugs retention in the stomach while protecting it against enzymatic degradation. It has attracted growing investigation due to its properties of biodegradability, biocompatibility, low toxicity, low immunogenicity and good mucoadhesion [24,25,26,27,28,29]. However, ALG microspheres have a loose network which results in the low encapsulation efficiency (EE) during preparation and low stability in gastric juice. To solve this problem, many researchers have been investigating the use of ALG microparticles coated with polycationic polymers of chitosan (CS) [30,31,32]. CS is a polysaccharide derived from the *N*-deacetylation of chitin, the second most abundant natural biopolymer [33]. Due to its good biocompatibility, biodegradability, nontoxicity, and significant adsorption and mucoadhesive properties, CS has been widely used in food, environmental, pharmaceutical nutraceutical industry recently [34,35,36,37,38]. Previous studies showed that CS/ALG microcapsule was efficient in protecting protein and peptide drugs from the invasive environment of the stomach and sustained protein and peptide drugs release [34,39].

In the study, in order to improve the PHLC-Ca bioavailability after its oral administration, firstly, PHLC-Ca-loaded microcapsules were prepared using ALG and CS as wall materials by the technology of impulse electrostatic droplet generation. Secondly, the characteristics of microcapsules were evaluated by laser particle size analyzer, scanning electron microscopy (SEM), Fourier transform-infrared spectroscopy (FT-IR) and Thermal gravimetric analysis (TGA). Thirdly, cell test and animal experiment were performed to investigate their security and bioactivity.

## 2. Materials and Methods

### 2.1. Materials

PHLC-Ca was prepared in our previous studies [17]. ALG was purchased from Sinopharm Chemical Reagent Co. Ltd. (Shanghai, China). CS (molecular weight: 100 kDa) was ordered from Jinke Biochemical Co. Ltd. (Hangzhou, China), and the degree of deacetylation is 91%. 3-(4,5-dimethylthiazol-2-yl)-2,5-diphenyl-2Htetrazolium bromide (MTT) was purchased from Sigma-Aldrich Co. (St. Louis, MO, USA). Other reagents used for the experimental procedures were of analytical grade and deionized water was used throughout.

### 2.2. Preparation of Microcapsules

**Figure 1:**
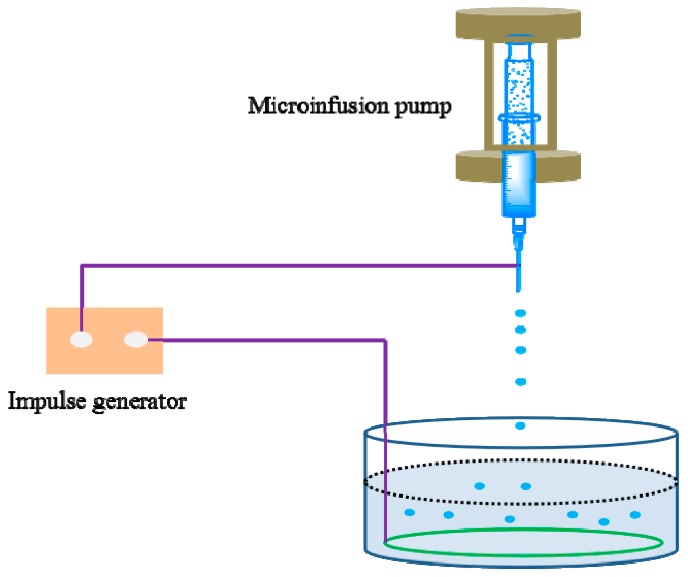
The impulse electrostatic device.

ALG-(PHLC-Ca) microspheres were produced by electrostatic droplet generation (Figure 1). ALG-(PHLC-Ca) intermixture was formed by mixing 15 mL of ALG (2%, *w*/*v*) solution with 5 mL of PHLC-Ca and the mass ratio of ALG to PHLC-Ca was 1:1, 1:1/2, 1:1/3, respectively. The intermixture was infused into a 20 mL plastic syringe connected to the anode of the impulse electrostatic device and extruded into a dish containing 50 mL of calcium chloride solution (2% *w*/*v*, pH = 5) (the needle diameter is 0.4 mm, the electrical potential is 380 V and the pulse frequency is 120 Hz).The divalent calcium ions crosslinked the droplets of sodium alginate to form loading PHLC-Ca of ALG microspheres (ALG-(PHLC-Ca)). The ALG-(PHLC-Ca) microspheres formed and were allowed to harden in a CaCl_2_ solution for 30 min and then were rinsed thrice with deionized water. The ALG-(PHLC-Ca) microspheres were transferred to the solution of CS (1% (*w*/*v*) in 1% (*v*/*v*) acetic acid) and shaked gently with shaker for 20 min to evenly coat the surface of the ALG-(PHLC-Ca) microspheres. The resulting CS/ALG-(PHLC-Ca) microcapsules were again separated by filtration and rinsed thrice with deionized water. They were frozen at −72 °C for 2 h in the freezer and lyophilized with a freeze drier for 24 h (FD 5–10, SIM, Charlotte, NC, USA).

### 2.3. Determination of Encapsulation Efficiency (EE) and Loading Capacity (LC)

Lyophilized samples were immersed in liquid nitrogen for 3 min and then broken off to detect the fracture surface. All prepared samples were gold coated and analyzed using scanning electron microscope (Hitachi S-570, Tokyo, Japan). The amount of PHLC-Ca loaded in microcapsules was measured as follows: CS/ALG-(PHLC-Ca) microcapsules (30 mg) were disintegrated in 6 mL 0.06 M sodium citrate–0.2 M sodium bicarbonate buffer solution and under vibration with the hand shake at ambient temperature. PHLC-Ca concentrations in homogenized samples were determined by the Micro BCA Protein Assay (Pierce Inc., New York, NY, USA) with PHLC-Ca as the standard protein.

The EE and LC of microcapsules was calculated using the following formulas (1) and (2) respectively:

(1) $$EE(\%)=\frac{X_{t}}{X_{i}}\times 100\%$$

(2) $$LC(\%)=\frac{X_{i}}{M}\times 100\%$$

where $X_{t}$ is the total amount of PHLC-Ca loaded in microcapsules, $X_{i}$ represents the initial amount of PHLC-Ca added in the preparation process, and M stands for weight of microcapsules.

### 2.4. Characterization of Microcapsules

#### 2.4.1. Particle Size Analysis

The particle size distribution of the microcapsules was analyzed using a laser particle size analyzer (Malvernpanalytical, London, UK). The dried microcapsules were loaded into deionized water and measurement was performed by static light scattering.

#### 2.4.2. Microcapsules Morphology

The surface morphology of the microcapsules was detected by scanning electron microscopy (SEM) (Model Hitachi S-570, Tokyo, Japan). Freeze dried microcapsules were fixed on SEM stubs using a double-sided tape and coated with gold metal under a high-vacuum evaporator. Sputter coated samples were then observed by SEM.

#### 2.4.3. Fourier Transform-Infrared Spectroscopy

Fourier transform infrared (FT-IR) analysis was carried out with a FT-IR spectrophotometer (Model Thermo Fisher Scientific, Waltham, MA, USA). All the samples were finely ground with KBr to prepare the pellets which were scanned from 4000 to 450 cm^−1^ at room temperature.

#### 2.4.4. Thermal Analysis

Thermal gravimetric analysis (TGA) was tested by thermogravimetric analyzer (Netzsch, Selb, Germany). The dried samples were placed in crucible with the temperature from 25 to 600 °C under nitrogen atmosphere and the heating rate of 10 °C/min.

#### 2.4.5. Confocal Laser Scanning Microscopy (CLSM)

Fluorescein isothiocyanate (FITC)-labelled CS was prepared as follows: 100 mL of CS solution (1% (*w*/*v*) in 2% (*v*/*v*) acetic acid) was regulated pH to 10–12 with 2 M NaOH. FITC (0.1 g/mL in PBS) was added to give a final FITC to CS ratio of 1:50. After stirring overnight at room temperature in the darkness, the mixture was collected by centrifuging and washed with PBS until the free FITC could not be detected in the supernatant. The resulting FITC-labelled CS was then frozen at −80 °C for 2 h in the freezer and lyophilized in a freeze drier (SIM, Charlotte, NC, USA) for 24 h in the dark.

FITC-labelled CS (1% *w*/*v*) was used to coat ALG microcapsules. The resulting samples were investigated using CLSM equipped with Argon (488 nm) and HeNe (534 nm) lasers. The microcapsules in storage solution (deionized H_2_O) were directly placed in the plastic plate and analyzed images were thus acquired.

### 2.5. Release Profiles of PHLC-Ca from Microcapsules In Vitro in the Gastrointestinal Environments

The 80 mg CS/ALG-(PHLC-Ca) microcapsules were dipped into 10 mL simulated gastric fluid (hydrochloric acid buffer at pH 2.0). The ratio of ALG to PHLC-Ca was 1:1/2. The fluid was incubated (37 °C, 100 rpm) for 2 h. At pre-determined time points, 100 μL of this solution was removed and separated from the microcapsules by centrifugation (3000 *g*/5 min) to obtain the supernatant for PHLC-Ca determination. The volume of the release medium was kept constant by replacing the withdrawn sample with an equal volume of fresh buffer. To simulate the process of microcapsules moving from the stomach into the intestine, after 2 h, the microcapsules were transferred to 10 mL simulated intestinal fluid (phosphate buffer at pH 7.4). The fluid was incubated (37 °C, 100 rpm) for 4 h. At determined times, 100 μL of supernatant was taken, separated from microcapsules by centrifugation (3000 *g*/5 min) for PHLC-Ca determination and replaced by fresh medium.

### 2.6. In Vitro Cell Cytotoxicity

The lyophilized samples were sterilized by ^60^Co irradiation and then placed into tubes with fresh culture medium added at 0.1 mg/mL, 0.5 mg/mL, 1 mg/mL, 10 mg/mL at 37 °C for 24 h. Mouse L929 fibroblast cells were seeded in wells of a 96-well at a density of 1.0 × 10^4^ cells/mL with 100 μL/well (*n* = 6). Cells were cultured at 37 °C in an incubator with a 5% CO_2_ and 95% air atmosphere at constant humidity in order to make cells attached to the bottom of the culture plate. After incubation for 24 h, the culture medium was removed and replaced with the extraction medium and incubated for 24 h, 36 h, 48 h. Cells in the control group were cultured with fresh medium. The viability of cells was determined by the MTT assay and about 50 μL of MTT solution was added to each well, after which the cultures were incubated at 37 °C for an additional 4 h, the culture medium and MTT solution were removed and replaced with 50 μL of DMSO. The optical density (OD) of the formazan solution was detected at 490 nm using an enzyme-linked immunosorbent assay (ELISA) plate reader (MODEL550, Bio-Rad, Berkeley, CA, USA). The relative cell growth was calculated using the following formula (3):

(3) $$\mathrm{Relative}\;\mathrm{cell}\;\mathrm{growth}=\frac{OD_{test}}{OD_{control}}\times 100\%$$

The mean value of six parallel samples was analyzed, and the whole test was repeated twice.

### 2.7. Determination of PHLC-Ca Bioavailability after Oral Administration of CS/ALG-(PHLC-Ca) Microcapsules

All animal care and experimental procedure were in accordance with the recommendations of the Regulations of the Administration of Affairs Concerning Experimental Animal and were approved by the Experimental Animal Centre at Northwest University. Call number is NWU201705244. Osteoporosis was induced in mice by being given a gavage of retinoic acid as described previously [40]. Forty male mice (4-week old, 22 ± 1.7 g) were randomly divided into two groups. Mice in the model group (*n* = 30) were given a gavage of retinoic acid (70 mg/kg/d) and fed low calcium diet for 2 weeks, mice in the control group (*n* = 10) were given a gavage of saline and fed a normal diet for 2 weeks. After 2 weeks, the model group was further randomly divided into one no supplement group (*n* = 5, Ca-deficiency group) and four calcium supplement groups (*n* = 5 each, Empty CS/ALG, PHLC-Ca, Col-Ca, CS/ALG-(PHLC-Ca). The ratio of ALG to PHLC-Ca was 1:1/2. Each group mice were given the normal diets and distilled water for the next 12 weeks.

Achieve experimentally determined time, mice were executed to obtain blood and bone samples. The serum was separated by centrifugation at 3000 rpm, for 10 min at 4 °C and used to determine calcium and alkaline phosphatase (ALP) levels. The bone samples were determined for hydroxyproline, calcium, bone density. The calcium in the serum and in the femurs were measured by atomic absorption spectrophotometry. The ALP and hydroxyproline were measured by ALP kit and hydroxyproline kit, respectively. The tibias were processed using a muffle furnace at 800 °C for 5 h and the fracture surface were detected by SEM.

### 2.8. Calculations and Data Analysis

The data were collected in a Microsoft Excel 2000 database, and the results are presented as the mean values and standard deviations using Origin 8.5 software (Originlab, Northampton, MA, USA). Student’s *t*-test was performed to determine the statistical significance between experimental groups. A value of *p* < 0.05 was considered to be statistically significant. A value of *p* ˂ 0.01 was considered to be highly significant.

## 3. Results and Discussion

### 3.1. Determination of Encapsulation Efficiency (EE) and Loading Capacity (LC)

The EE and LC of formulations with different ratios were calculated and the data were shown in Table 1. With the increasing of PHLC-Ca, the EE tended to decrease. But when the mass ratio was 1:1/2, the LC change was almost unchanged. It may be due to the surplus of PHLC-Ca. So 1:1/2 was the best ratio of microcapsules.

### 3.2. Characterization of Microcapsules

#### 3.2.1. Morphology of Microcapsules

**Figure 2:**
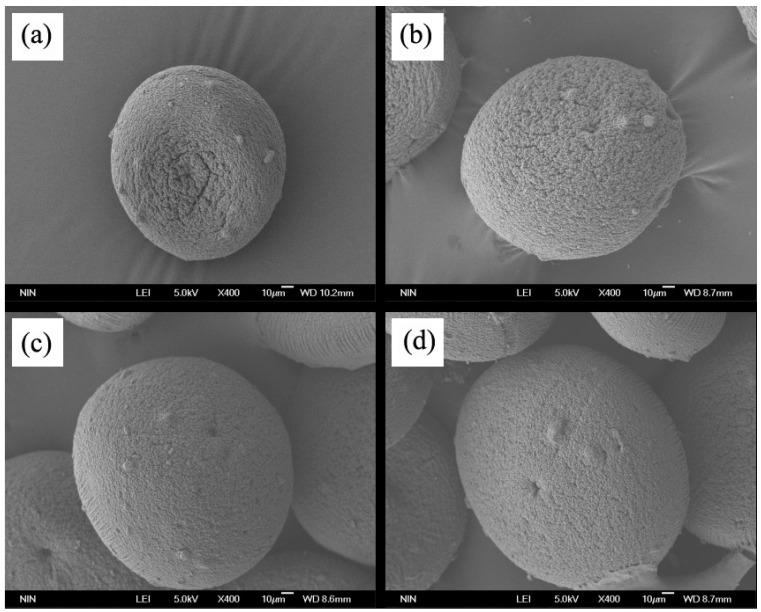
Scanning electron micrograph of microparticles. (**a**) Empty CS/ALG; (**b**) CS/ALG-(PHLC-Ca)(ALG:PHLC-Ca1:1/3); (**c**) CS/ALG-(PHLC-Ca)(ALG:PHLC-Ca1:1/2); (**d**) CS/ALG-(PHLC-Ca)(ALG:PHLC-Ca 1:1).

The macroscopic features of empty CS/ALG microcapsules and all CS/ALG-(PHLC-Ca) microcapsules were characterized by SEM (Figure 2). Empty CS/ALG microcapsules (Figure 2a) and CS/ALG-(PHLC-Ca) microcapsules with the proportion of 1:1/3 (Figure 2b) showed partial collapse and had cracks on microcapsules surface, but CS/ALG-(PHLC-Ca) microcapsules with the proportion of 1:1/2 (Figure 2c) and 1:1 (Figure 2d) were generally spherical during the freeze-drying process due to high level of PHLC-Ca and was more plump than that of the empty microcapsules and CS/ALG-(PHLC-Ca) microcapsules with the proportion of 1:1/3.

#### 3.2.2. Size Distribution and CLSM of Microcapsules

**Figure 3:**
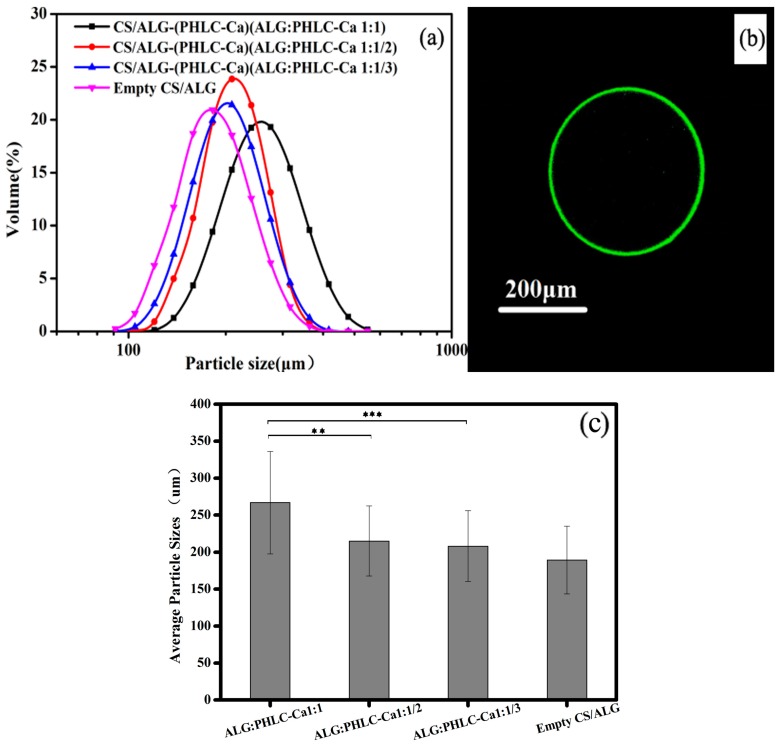
(**a**) Particle size distribution curves of microcapsules; (**b**) confocal laser scanning microscopy (CLSM) images of chitosan (CS) coated alginate (ALG) microcapsules; (**c**) Average particle sizes of microcapsules. ** *p* < 0.01; *** *p* < 0.001.

Figure 3a shows the particle size distribution curves of microcapsules. Figure 3c shows the average particle of the empty microcapsules and CS/ALG-(PHLC-Ca) microcapsules with the proportion of 1:1, 1:1/2 and 1:1/3, 1:1/3 of the CS/ALG-(PHLC-Ca) microcapsules were extremely significant compared to 1:1 of the CS/ALG-(PHLC-Ca) microcapsules, 1:1 of the CS/ALG-(PHLC-Ca) microcapsules being very significant compared to 1:1/2 of the CS/ALG-(PHLC-Ca) microcapsules. As the PHLC-Ca ratio decreases, the particle size of the microcapsules also decreases.

The CLSM was employed to visualize the microcapsules and their membranes. Figure 3b depicts confocal microscopy images of 1% (*w*/*v*) FITC-labelled CS coated ALG microcapsules. The microcapsule shells were clearly visible on the optical microscopy images and on fluorescence microscopy images where the FITC-labelled CS coat appeared green whereas the ALG core was observed as black. This indicated that CS were absorbed on ALG microcapsules.

#### 3.2.3. FT-IR Spectroscopic Analysis

**Figure 4:**
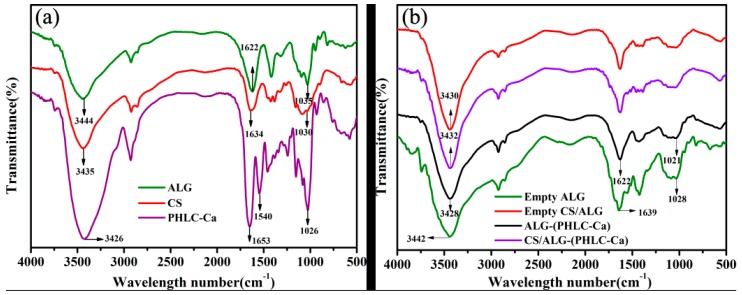
Fourier transform infrared (FTIR) spectra of (**a**) ALG, CS, PHLC-Ca; (**b**) Empty ALG, Empty CS/ALG, ALG-PHLC-Ca and CS/ALG-(PHLC-Ca) microcapsules.

Figure 4 shows the FTIR spectra for ALG, CS, PHLC-Ca, Empty ALG, Empty CS/ALG, ALG-PHLC-Ca and CS/ALG-(PHLC-Ca) microcapsules. As shown in the spectrum of ALG (Figure 4a), the peaks around 3444, 1622 and 1035 cm^−1^ were the stretching of OH, COO– (asymmetric), and C–O–C, respectively. For the empty ALG microcapsules, crosslinking of ALG by Ca^2+^ caused an obvious shift to higher wave number of COO– stretching peak at 1639 cm^−1^ (Figure 4b) and a decrease in COO– stretching peaks intensity, indicating ionic bonding between calcium ion and carboxyl groups of ALG. Moreover, the stretching peak of C–O–C at 1035 cm^−1^ (Figure 4a) shifted to 1028 cm^−1^ (Figure 4b), owing to partial covalent bonding between calcium and oxygen atom [41]. ALG-PHLC-Ca microcapsules shown an obvious decrease in the wave number from 1639 to 1622 cm^−1^ and C–O–C stretching peak at 1028 cm^−1^ shifted to 1021 cm^−1^ (Figure 4b). Meanwhile, the greater intensity peak of OH stretching of the ALG-(PHLC-Ca) microcapsules showed lower wavenumber at around 3428 cm^−1^ than the empty ALG microcapsules [42]. Owing to intermolecular hydrogen bonding and electrostatic force interaction between C=O, O–H and N–H of ALG and PHLC-Ca. The above phenomena demonstrated that ALG and PHLC-Ca in ALG-(PHLC-Ca) microcapsules could form intermolecular hydrogen bonding and electrostatic force. As shown in the spectrum of CS (Figure 4a). The characteristic peaks were at 3435 cm^−1^ (O–H stretching vibration and N–H extension vibration), 1634 cm^−1^ (amide C=O stretching), 1030 cm^−1^ (C–O–C stretching). The OH stretching peak at 3430 cm^−1^ of the empty CS/ALG had a narrower peak than that of empty ALG. Meanwhile, ALG-(PHLC-Ca) and CS/ALG-(PHLC-Ca) had the same phenomenon. This should be attributed to CS coated on ALG microcapsules.

#### 3.2.4. Thermal Analysis

**Figure 5:**
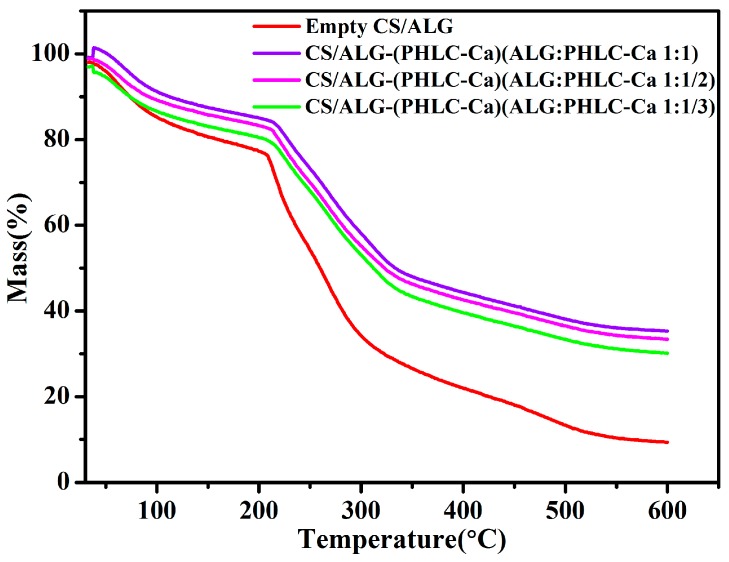
Thermal gravimetric analysis (TGA) curves of Empty CS/ALG and CS/ALG-(PHLC-Ca) microcapsules.

A comparison of the loading amount for the CS/ALG-(PHLC-Ca) microcapsules was further evaluated based on the TGA results. The decrement of mass loss could be considered as a reliable indicator of the increasing loading amount. Figure 5 shows the TGA curves of the empty CS/ALG microcapsules and all the CS/ALG-(PHLC-Ca) microcapsules. The first weight loss before 130 °C was attributed to the weight loss of water in the samples [43]. The gradual mass loss was attributed to thermal degradation of the materials, as shown in Figure 4 in the range of 130 to 600 °C. The weight loss of the empty CS/ALG microcapsules was about 72.88% when the temperature was 600 °C. After loading PHLC-Ca, the weight loss of the CS/ALG-(PHLC-Ca) microcapsules with different ratios of ALG to PHLC-Ca (ALG:PHLC-Ca 1:1, 1:1/2, 1:1/3) between the temperatures of 130 to 600 °C decreased to 55.70%, 56.59%, 59.89%, respectively. This indicated that PHLC-Ca was successfully loaded in the CS/ALG-(PHLC-Ca) microcapsules via intermolecular hydrogen bonding and electrostatic force, and LC of the CS/ALG-(PHLC-Ca) microcapsules increased with the increasing of PHLC-Ca.

### 3.3. Release Profile In Vitro

**Figure 6:**
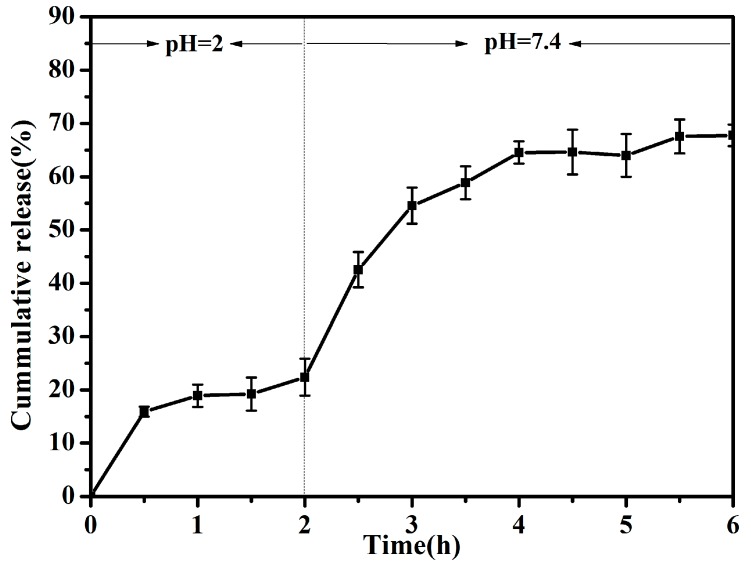
The release profile of PHLC-Ca in vitro in the gastrointestinal environments.

The PHLC-Ca release behavior from the CS/ALG-(PHLC-Ca) microcapsules in vitro in the gastrointestinal environments was shown in Figure 6. As observed, the only about 18% PHLC-Ca released from the microcapsules after 2 h in the simulating gastric condition (pH 2.0). Then the microcapsules were transferred to simulated intestinal fluid (pH 7.4), where a sustained and prolonged PHLC-Ca release was observed freeing about 68% of the initial amount. The observed little amount release of PHLC-Ca at pH 2 could be attributed to CS shell and tight alginate network that formed at low pH, this indicated that the microcapsules would protect the encapsulated PHLC-Ca from the lesion of the low pH and proteolytic enzymes in stomach. Moreover, the sustained and prolonged PHLC-Ca release from CS/ALG-(PHLC-Ca) microcapsules at pH 7.4 was achieved, owing to the swelling of ALG microcapsules at an alkaline pH and the penetration of the alkaline solvent towards the ALG microcapsules core. Therefore, the microcapsules were efficient in protecting the PHLC-Ca in stomach minimizing the PHLC-Ca loss in the gastrointestinal tract.

Gim-Pao Lim, Muhammad Syarhabil Ahmad prepared Ca-alginate-chitosan microcapsules for encapsulation and controlled release of imidacloprid [44], release results show that, no matter how the concentration of chitosan or sodium alginate was changed, the temperature and pH were adjusted, after 20 h, the release rate in vitro would not exceed 60%, even lower. The release rates in vitro of Chitosan (CS)-microcapsules (MCs) and Antofloxacin-loaded CS-MCs and starch (AMS) films prepared by Weiqiang Huo and Gancheng Xie were 70% and 15%. The release rates in vitro after 20 h were 95% and 30%, after 5 h were 52% and 8% respectively [45]. For microcapsules, excessively long release times and excessive release rates are detrimental, especially drugs or supplements, time of digestion and absorption of the human body does not exceed 6 h, the small intestine is the most important place of absorption, so the drug encapsulated in the microcapsules to release in a large amount at specific time. Therefore, microcapsules in the simulated gastrointestinal fluid in vitro environment, to ensure that the drug release between 3 h to 5 h to achieve the best absorption will be a major challenge of microcapsule sustained release.

### 3.4. MTT Assay

**Figure 7:**
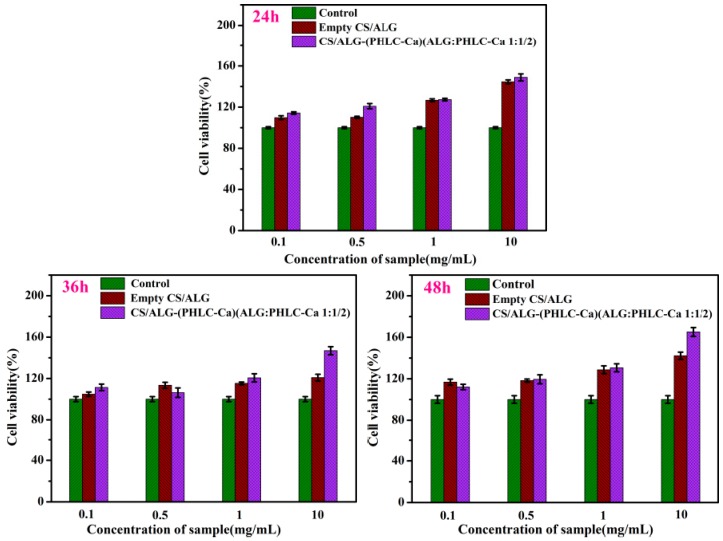
Proliferation graph of the mouse L929 fibroblast cells cultured with different concentrations of microcapsules extracts for 24, 36 and 48 h, analyzed by the MTT assay.

The MTT assay is generally accepted as a routine method for establishing the toxicity of different substances in cell cultures. The cytotoxicity of the microspheres extracts (extracts concentration are 0.1, 0.5, 1, 10 mg/mL) was assessed by MTT assay for 24 h, 36 h and 48 h (Figure 7). Empty CS/ALG microcapsules could promote mouse L929 fibroblast cells proliferation, and following the increase of concentration of empty CS/ALG microcapsules extracts, the mouse L929 fibroblast cells proliferation was promoted obviously. Compared with the control, CS/ALG-(PHLC-Ca) microcapsules extracts triggered a significant increase in the number of the mouse L929 fibroblast cells at 24 h, 36 h and 48 h. Therefore, empty CS/ALG microcapsules and CS/ALG-(PHLC-Ca) microspheres were safe to the mouse L929 fibroblast cells and could stimulate the normal growth of the mouse L929 fibroblast cells, which could be a safe polymeric carrier for oral PHLC-Ca delivery.

### 3.5. Animal Experiments

**Figure 8:**
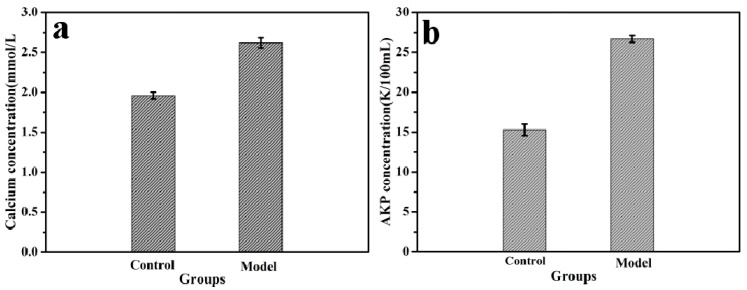
Calcium concentrations (**a**) and alkaline phosphatase (ALP) concentrations (**b**) in the serum of mice. Model groups and Control groups.

The calcium and ALP content in the serum for 2 weeks are shown in Figure 8. Compared with the control group, the serum calcium and ALP levels of the model group were significantly increased. This should be attributed to the overdose of retinoic acid for a long time that led to the inhibition of osteoblasts and the activity of osteoclast, resulting in the increase of the dissolution of bone calcium and the blood calcium content. In addition, the diseased bone caused ALP released into the blood. This indicated that the osteoporosis model on mice was successfully established.

**Figure 9:**
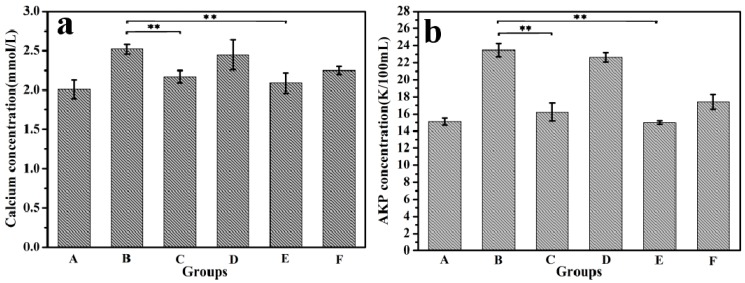
Calcium concentrations (**a**) and ALP concentrations (**b**) in the serum of osteoporosis mice which supplement calcium for 12 weeks. A, Control group; B, Ca-deficiency group; C, PHLC-Ca group; D, Empty CS/ALG group; E, CS/ALG-(PHLC-Ca) group; F, Col-Ca group. ** *p* < 0.01.

After 14 weeks, the serum calcium and ALP content in the CS/ALG-(PHLC-Ca) group, PHLC-Ca group and Col-Ca group showed a significantly decreased compared with in the Ca-deficiency group (Figure 9). However, the ALP and calcium levels in the CS/ALG-(PHLC-Ca) group was closer to the normal levels than those in the PHLC-Ca group and Col-Ca group. This indicated that PHLC-Ca bioavailability was increased in CS/ALG-(PHLC-Ca) microcapsules.

**Figure 10:**
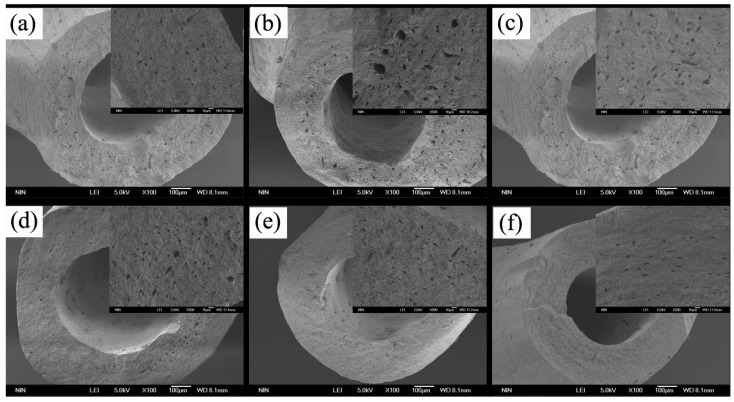
SEM images of the section of tibia from osteoporosis mice that supplement calcium for 12 weeks. (**a**) Control group; (**b**) Ca-deficiency group; (**c**) Empty CS/ALG group; (**d**) Col-Ca group; (**e**) PHLC-Ca group; (**f**) CS/ALG-(PHLC-Ca) group.

Figure 10 shows the scanning electron microscopy image of the section of mice tibia. Size and number of holes indirectly reflect bone quality. As shown in Figure 10, a greater number and larger size of holes were observed in the Ca-deficiency group than in the control group. The holes of other experimental groups were fewer and smaller than the Ca-deficiency group. The least and smallest holes were observed in the CS/ALG-(PHLC-Ca) group. Thus, administering CS/ALG-(PHLC-Ca) microcapsules to osteoporotic mice resulted in higher bone mineral density of the tibia than the mice given other supplements. Compared with PHLC-Ca group, Calcium concentrations and ALP concentrations in CS/ALG-(PHLC-Ca) group were closer to the serum calcium level and serum alkaline phosphatase of control group, but there was no significant difference between CS/ALG-(PHLC-Ca) and PHLC-Ca groups.

**Figure 11:**
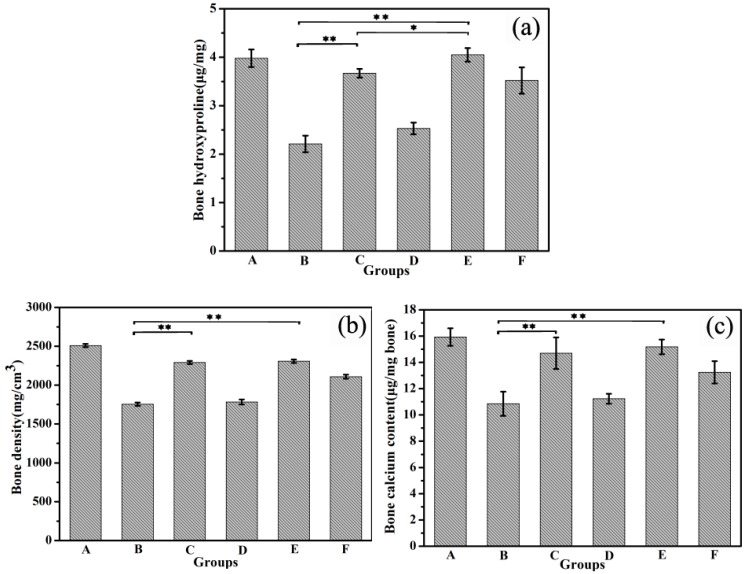
Bone hydroxyproline content (**a**) Bone density (**b**) and Bone calcium content of osteoporosis mice that supplement calcium for 12 weeks (**c**). A, Control group; B, Ca-deficiency group; C, PHLC-Ca group; D, Empty CS/ALG group; E, CS/ALG-(PHLC-Ca) group; F, Col-Ca group. * *p* < 0.05; ** *p* < 0.01.

Collagen I accounts for 90% of the organic components of the bone, which can reflect the quality of the bone. Therefore, hydroxyproline of the main amino acid of collagen I indirectly determines the quality of the bone. Compared with the Ca-deficiency group, the hydroxyproline content in the right femur was improved in different degrees in the PHLC-Ca group, CS/ALG-(PHLC-Ca) group, and Col-Ca group (Figure 11a). However, the CS/ALG-(PHLC-Ca) group showed higher hydroxyproline content than the other groups. This indicated that the osteoporosis was improved, and the improvement of the CS/ALG-(PHLC-Ca) group was the most obvious.

Bone densities, as one of the important standards of bone quality, has become one of the important evaluation means of osteoporosis in the clinical judgment. As shown in Figure 11b, the bone densities of the empty CS/ALG group were not significantly different from those of the Ca-deficiency group. The mice in Col-Ca group had lower bone densities than the mice treated with PHLC-Ca and CS/ALG-(PHLC-Ca). However, the bone densities of the CS/ALG-(PHLC-Ca) group were the closest to that of the control group. This indicated that CS/ALG-(PHLC-Ca) had higher bioavailability than the PHLC-Ca group.

As shown in Figure 11c, the calcium content in the left femur was increased in the Col-Ca group, the PHLC-Ca group and the CS/ALG-(PHLC-Ca) group compared with the Ca-deficiency group, which suggested that calcium had precipitation in the bone. The highest calcium content was observed in the CS/ALG-(PHLC-Ca) group. Compared with PHLC-Ca group and Col-Ca group, bone calcium content and bone density in CS/ALG-(PHLC-Ca) group were closest to the bone calcium content and bone density of control group, there was no significant difference between the CS/ALG-(PHLC-Ca) group and PHLC-Ca group. The content of bone hydroxyproline in CS/ALG-(PHLC-Ca) group was the highest, which was significantly different from that of PHLC-Ca group. Although the significance of each index was not significant, the effects of CS/ALG-(PHLC-Ca) have increased in varying degrees, indicating that the Phosphorylated Human-like Collagen-calcium chelates microencapsulation is an effective method to increase the bioactivity and bioavailability.

## 4. Conclusions

Encapsulation is critical to improve bioavailability of protein drugs in oral administration. In this study, the CS/ALG-(PHLC-Ca) microcapsules was successfully prepared using of ALG and CS as wall materials and further evaluated for protection and controlled releasing of the PHLC-Ca. The particle size distribution and SEM images showed that the microcapsules had spherical morphologies. The characteristics of microcapsules were confirmed by FT-IR and TGA experiments. These data indicated that PHLC-Ca entrapped into the matrix of ALG through forming intermolecular hydrogen bonding or other interactions. The cell viability study confirmed that the prepared CS/ALG-(PHLC-Ca) microcapsules were non-toxic. The animal experiment showed that CS/ALG-(PHLC-Ca) microcapsules was superior to PHLC-Ca for treating osteoporosis. Finally, the bioavailability of PHLC-Ca was improved by CS/ALG-(PHLC-Ca) microcapsules, which would provide new ideas for the future development and research of proteins calcium supplements.

## Figures and Tables

**Table 1:** The Encapsulation Efficiency (EE) and Loading Capacity (LC) of microcapsules with different ratios of ALG to PHLC-Ca.

ALG:PHLC-Ca (*w*:*w*)	EE (%)	LC (%)
1:1/3	53.70 ± 0.97%	13.42 ± 0.81%
1:1/2	44.05 ± 1.69%	16.31 ± 0.96%
1:1	25.41 ± 1.02%	16.54 ± 0.58%

## References

[1] Power M.L., Heaney R.P., Kalkwarf H.J. (1999). The role of calcium in health and disease. Am. J. Obstet. Gynecol..

[2] Cho E., Smith-Warner S.A., Spiegelman D. (2004). Dairy foods, calcium, and colorectal cancer: A pooled analysis of 10 cohort studies. J. Natl. Cancer Inst..

[3] Vavrusova M., Skibsted L.H. (2014). Calcium nutrition. Bioavailability and fortification. LWT Food Sci. Technol..

[4] Ribeiro A.C., Rita M.B., Gomes J.C., Lobo V.M., Esteso M.A. (2011). Diffusion of calcium gluconate in aqueous solutions of lactose at 298.15 K. Food Chem..

[5] Chen D., Mu X., Huang H., Nie R., Liu Z., Zeng M. (2014). Isolation of a calcium-binding peptide from tilapia scale protein hydrolysate and its calcium bioavailability in rats. J. Funct. Foods.

[6] Schinke T., Schilling A.F., Baranowsky A. (2009). Impaired gastric acidification negatively affects calcium homeostasis and bone mass. Nat. Med..

[7] Touati G., Valayannopoulos V., Mention K. (2006). Methylmalonic and propionic acidurias: Management without or with a few supplements of specific amino acid mixture. J. Inherit. Metab. Dis..

[8] Jung W.K., Karawita R., Heo S.J., Lee B.J., Kim S.K., Jeon Y.J. (2006). Recovery of a novel Ca-binding peptide from Alaska Pollack (*Theragra chalcogramma*) backbone by pepsinolytic hydrolysis. Process Biochem..

[9] Bass J.K., Chan G.M. (2006). Calcium nutrition and metabolism during infancy. Nutrition.

[10] Fairweather-Tait S.J., Teucher B. (2002). Calcium bioavailability in relation to bone health. Int. J. Vitam. Nutr. Res..

[11] Sugiarto M., Ye A., Singh H. (2009). Characterisation of binding of iron to sodium caseinate and whey protein isolate. Food Chem..

[12] Xiu F.H., Daidi F., Zhang X. (2006). Kinetics of high cell density fed-batch culture of recombinant *Escherichia coli* producing human-like collagen. Chin. J. Chem. Eng..

[13] Guo J., Luo Y., Fan D. (2010). Medium optimization based on the metabolic-flux spectrum of recombinant *Escherichia coli* for high expression of human-like collagen II. Biotechnol. Appl. Biochem..

[14] Zhang C., Daidi F., Shang L., Xiaoxuan M., Wenjiao X., Pengfei G. (2010). Optimization of fermentation process for human-like collagen production of recombinant *Escherichia coli* using response surface methodology. Chin. J. Chem. Eng..

[15] Zhai Y., Cui F. (2006). Recombinant human-like collagen directed growth of hydroxyapatite nanocrystals. J. Cryst. Growth.

[16] Zhu C., Fan D., Duan Z. (2009). Initial investigation of novel human-like collagen/chitosan scaffold for vascular tissue engineering. J. Biomed. Mater. Res. A.

[17] Zhu C., Chen Y., Deng J. (2015). Preparation, characterization, and bioavailability of a phosphorylated human-like collagen calcium complex. Polym. Adv. Technol..

[18] Sood A., Panchagnula R. (2001). Peroral route: An opportunity for protein and peptide drug delivery. Chem. Rev..

[19] Zhang Y., Wei W., Lv P., Wang L., Ma G. (2011). Preparation and evaluation of alginate–chitosan microspheres for oral delivery of insulin. Eur. J. Pharm. Biopharm..

[20] Dai C., Wang B., Zhao H. (2005). Microencapsulation peptide and protein drugs delivery system. Colloids Surf. B Biointerfaces.

[21] Yeo Y., Baek N., Park K. (2001). Microencapsulation methods for delivery of protein drugs. Biotechnol. Bioprocess Eng..

[22] Tran V.T., Benoît J.P., Venier-Julienne M.C. (2011). Why and how to prepare biodegradable, monodispersed, polymeric microparticles in the field of pharmacy?. Int. J. Pharm..

[23] Ma G. (2014). Microencapsulation of protein drugs for drug delivery: Strategy, preparation, and applications. J. Control. Release.

[24] Martins A.F., Bueno P.V., Almeida E.A., Rodrigues F.H., Rubira A.F., Muniz E.C. (2013). Characterization of *N*-trimethyl chitosan/alginate complexes and curcumin release. Int. J. Biol. Macromol..

[25] Chan L., Jin Y., Heng P. (2002). Cross-linking mechanisms of calcium and zinc in production of alginate microspheres. Int. J. Pharm..

[26] Silva C.M., Ribeiro A.J., Figueiredo I.V., Gonçalves A.R., Veiga F. (2006). Alginate microspheres prepared by internal gelation: Development and effect on insulin stability. Int. J. Pharm..

[27] Sundar S., Kundu J., Kundu S.C. (2010). Biopolymeric nanoparticles. Sci. Technol. Adv. Mater..

[28] Vrignaud S., Benoit J.P., Saulnier P. (2011). Strategies for the nanoencapsulation of hydrophilic molecules in polymer-based nanoparticles. Biomaterials.

[29] Azevedo M.A., Bourbon A.I., Vicente A.A., Cerqueira M.A. (2014). Alginate/chitosan nanoparticles for encapsulation and controlled release of vitamin B_2_. Int. J. Biol. Macromol..

[30] Lee J., Cha D., Park H.J. (2004). Survival of freeze-dried *Lactobacillus bulgaricus* KFRI 673 in chitosan-coated calcium alginate microparticles. J. Agric. Food Chem..

[31] Krasaekoopt W., Bhandari B., Deeth H.C. (2006). Survival of probiotics encapsulated in chitosan-coated alginate beads in yoghurt from UHT-and conventionally treated milk during storage. LWT Food Sci. Technol..

[32] De Vos P., Faas M.M., Strand B., Calafiore R. (2006). Alginate-based microcapsules for immunoisolation of pancreatic islets. Biomaterials.

[33] Shi C., Zhu Y., Ran X., Wang M., Su Y., Cheng T. (2006). Therapeutic potential of chitosan and its derivatives in regenerative medicine. J. Surg. Res..

[34] Sarmento B., Ribeiro A., Veiga F., Ferreira D., Eufeld R.N. (2007). Insulin-loaded nanoparticles are prepared by alginate ionotropic pre-gelation followed by chitosan polyelectrolyte complexation. J. Nanosci. Nanotechnol..

[35] Ho T.H., Le T.N.T., Nguyen T.A., Dang M.C. (2015). Poly (ethylene glycol) grafted chitosan as new copolymer material for oral delivery of insulin. J. Nanosci. Nanotechnol..

[36] Nagpal K., Singh S.K., Mishra D.N. (2010). Chitosan nanoparticles: A promising system in novel drug delivery. Chem. Pharm. Bull..

[37] Peniche C., Argüelles M.W., Peniche H., Acosta N. (2003). Chitosan: An attractive biocompatible polymer for microencapsulation. Macromol. Biosci..

[38] Mi Y., Su R., Fan D.D., Zhu X.L., Zhang W.N. (2013). Preparation of *N*,*O*-carboxymethyl chitosan coated alginate microcapsules and their application to *Bifidobacterium longum* BIOMA 5920. Mater. Sci. Eng. C.

[39] Mukhopadhya P., Chakrabort S., Bhattacharya S., Mishra R., Kundu P. (2015). pH-sensitive chitosan/alginate core-shell nanoparticles for efficient and safe oral insulin delivery. Int. J. Biol. Macromol..

[40] Wang A., Ding X., Sheng S., Yao Z. (2008). Retinoic acid inhibits osteogenic differentiation of rat bone marrow strom al cells. Biochem. Biophys. Res. Commun..

[41] Angadi S.C., Manjeshwar L.S., Aminabhav T.M. (2012). Novel composite blend microbeads of sodium alginate coated with chitosan for controlled release of amoxicillin. Int. J. Biol. Macromol..

[42] Wong T.W., Chan L.W., Kho S.B., Heng P.W.S. (2002). Design of controlled-release solid dosage forms of alginate and chitosan using microwave. J. Control. Release.

[43] Martins A.F., Facchi S.P., Monteiro J.P. (2015). Preparation and cytotoxicity of *N*,*N*,*N*-trimethyl chitosan/alginate beads containing gold nanoparticles. Int. J. Biol. Macromol..

[44] Lim G.P., Ahmad M.S. (2017). Development of Ca-alginate-chitosan microcapsules for encapsulation and controlled release of imidacloprid to control dengue outbreaks. J. Ind. Eng. Chem..

[45] Huo W., Xie G., Zhang W. (2016). Preparation of a novel chitosan-microcapsules/starch blend film and the study of its drug-release mechanism. Int. J. Biol. Macromol..

